# Identification and Structural Modeling of the RNA Polymerase Omega Subunits in Chlamydiae and Other Obligate Intracellular Bacteria

**DOI:** 10.1128/mbio.03499-22

**Published:** 2023-01-31

**Authors:** Andrew Cheng, Danny Wan, Arkaprabha Ghatak, Chengyuan Wang, Deyu Feng, Joseph D. Fondell, Richard H. Ebright, Huizhou Fan

**Affiliations:** a Department of Pharmacology, Rutgers-Robert Wood Johnson Medical School, Piscataway, New Jersey, USA; b Graduate Program in Physiology and Integrative Biology, Rutgers School of Graduate Studies, Piscataway, New Jersey, USA; c Center for Microbes, Development and Health, CAS Key Laboratory of Molecular Virology and Immunology, Institute Pasteur of Shanghai, Chinese Academy of Sciences, Shanghai, China; d Waksman Institute, Rutgers University, Piscataway, New Jersey, USA; e Department of Chemistry and Chemical Biology, Rutgers University, Piscataway, New Jersey, USA; Pennsylvania State University

**Keywords:** *Chlamydia*, RNA polymerases, omega subunit

## Abstract

Gene transcription in bacteria is carried out by the multisubunit RNA polymerase (RNAP), which is composed of a catalytic core enzyme and a promoter-recognizing σ factor. The core enzyme comprises two α subunits, one β subunit, one β′ subunit, and one ω subunit. The ω subunit plays critical roles in the assembly of the core enzyme and other cellular functions, including the regulation of bacterial growth, the stress response, and biofilm formation. However, the identity of an ω subunit for the obligate intracellular bacterium Chlamydia has not previously been determined. Here, we report the identification of the hypothetical protein CTL0286 as the probable chlamydial ω subunit based on sequence, synteny, and AlphaFold and AlphaFold-Multimer three-dimensional-structure predictions. Our findings indicate that CTL0286 functions as the missing ω subunit of chlamydial RNAP. Our extended analysis also indicates that all obligate intracellular bacteria have ω orthologs.

## INTRODUCTION

RNA synthesis in bacteria is carried by a single RNA polymerase (RNAP). The bacterial RNAP is a multisubunit enzyme (1). In almost all bacteria, the catalytic core enzyme of the RNAP (RNAP core) is composed of two α subunits, one β subunit, one β′ subunit, and one ω subunit (1, 2). The association of a σ factor with the core enzyme results in the formation of the RNAP holoenzyme (1). In the context of the holoenzyme, the σ factor is the primary determinant of promoter recognition and binding, and the RNAP core catalyzes the initiation and elongation of RNA synthesis using DNA as the template (2–4).

The RNAP ω subunit, a protein of only about 10 kDa, was initially thought to be a contaminant in purified RNAP preparations (5–7). This view was prompted by the observation that ω-free RNAP preparations were active in transcription assays (8). However, the observation of increased transcription initiation activity by RNAP derivatives having ω fused to DNA-binding domains indicated that ω was an integral component of RNAP (9). Further studies showed that ω is critical for the folding of the RNAP β′ subunit and the assembly and stability of the RNAP core enzyme (10–14). Studies using ω-deficient bacteria showed that ω is important for the response to amino acid starvation, thermal and CO_2_ acclimation, biofilm formation, and antibiotic production, and also affects growth under standard culture conditions (15–20). It was also shown that ω regulates the association of principal and alternative σ factors with the RNAP core enzyme and thus can affect promoter recognition selectivity (21–23). Taken together, these and other studies suggest that ω serves as an important component of the bacterial RNAP holoenzyme and is required for numerous physiological functions (for reviews, see references 24–26).

ω has been found in all free-living bacteria and some obligate intracellular bacteria (24, 25). ω is also present in some eukaryotic chloroplasts (27). An ortholog of ω termed RpoK is present in archaeal RNAP (28), and an ortholog of ω termed RPB6 is present in eukaryotic RNAP I, II, and III (29).

Chlamydiae are intracellular bacteria that replicate only inside eukaryotic host cells (30, 31). Chlamydiae and Chlamydia-like organisms have been isolated from a wide range of hosts (32–46). Significantly, Chlamydia trachomatis is the number-one sexually transmitted bacterial pathogen globally and is also a major cause of preventable blindness in developing countries (47–49), and Chlamydia pneumoniae is a common respiratory pathogen (50–54). Several animal Chlamydia species are zoonotic pathogens (55–64). Waddlia chondrophila is one of several Chlamydia-like organisms termed environmental chlamydiae that typically are found in lower eukaryotes such as amoebae but can infect, and induce abortion in, vertebrates, including humans (65).

Chlamydiae are characterized by a unique developmental cycle consisting of two distinct cellular forms. The infectious but nonproliferative elementary body (EB) is capable of temporarily surviving in extracellular environments and invading host cells. Following the invasion of host cells and entry into cytoplasmic vacuoles, EBs differentiate into proliferative reticulate bodies (RBs). Following multiple rounds of replication, RBs convert back into EBs, which then exit host cells (66–68). In addition to this “productive” chlamydial developmental cycle, under unfavorable environmental conditions (e.g., nutrient/mineral starvation, increased temperature, or exposure to inhibitory antibiotics or cytokines), chlamydiae can enter into a “persistent” state characterized by aberrant RBs inside infected cells, and when environmental conditions improve, the aberrant RBs can exit the persistent state and resume the production of EBs (69–75).

Both the productive chlamydial developmental cycle and persistent infection are controlled by gene transcription (66, 69, 71, 75, 76). The chlamydial genome encodes three σ factors (σ^66^, σ^28^, and σ^54^) as well as the α, β, and β′ subunits of the core enzyme. Surprisingly, it was not previously possible to identify a candidate gene encoding the ω subunit in any chlamydial genome (e.g., see references 77–80). In principle, the chlamydial *rpoZ* gene may have been lost in the evolutionary process during which Chlamydia reduced its genome size to adapt to its unique developmental cycle. Alternatively, in principle, the chlamydial ω protein may have gone undetected due to low sequence homology with known bacterial and chloroplast ω factors.

Here, we report the identification of chlamydial ω based on conserved amino acid sequence, conserved synteny, and AlphaFold-predicted conserved three-dimensional structure and interactions. In addition, we also present an AlphaFold-Multimer model of the three-dimensional structure of a complex composed of the chlamydial RNAP β, β′, and ω subunits. The identification of the previously elusive chlamydial ω sets the stage for investigations of its roles in the regulation of gene expression during chlamydial growth, development, and stress responses. Our findings also set the stage to reconstitute the intact chlamydial RNAP from recombinant subunits *in vitro* for future structural studies and for the discovery and development of small-molecule inhibitors as possible antichlamydial drugs.

## RESULTS

### Identification of chlamydial ω: sequence similarity.

**FIG 1:**
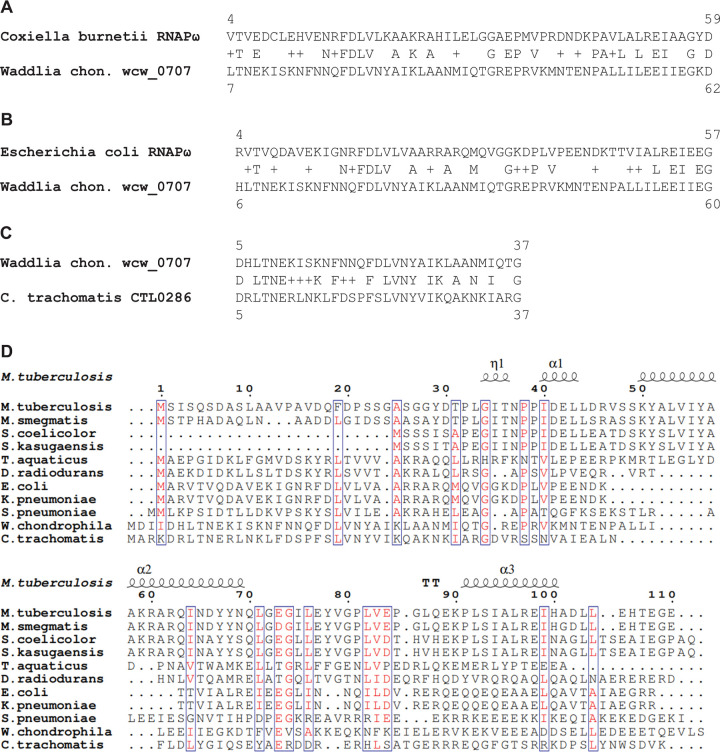
Identification of the cRNAP ω candidate by BLASTP analysis and sequence alignment. (A) BLASTP-detected sequence homology between the Coxiella burnetii RNAP ω subunit and Wcw_0707, a hypothetical protein of the Chlamydia-like organism Waddlia chondrophila (*Waddlia chon*.). (B) BLASTP-detected sequence homology between E. coli RNAP ω and Wcw_0707. (C) BLASTP-detected sequence homology between Wcw_0707 and CTL0286 of Chlamydia trachomatis. (D) ClustalX2-detected amino acids conserved in CTL0286 of C. trachomatis, Wcw_0707 of W. chondrophila, and ω subunits of a variety of bacteria. M. smegmatis, Mycobacterium smegmatis; S. coelicolor, Streptomyces coelicolor; *S. kasugaensis*, Streptomyces kasugaensis; *T. aquaticus*, *Thermus aquaticus*; D. radiodurans, Deinococcus radiodurans; K. pneumoniae, Klebsiella pneumoniae; S. pneumoniae, *Streptococcus pneumoniae*.

Although ω has not been detected in chlamydiae, ω had been detected in two other intracellular bacteria, *Rickettsia* and *Coxiella* (81, 82). Therefore, as a starting point to determine if chlamydiae encode an ω subunit, we performed a BLASTP analysis of chlamydial genomes using the amino acid sequences of Rickettsia rickettsii ω and Coxiella burnetii ω (81, 82) as queries. Using default parameters (83), the analysis did not detect sequences homologous to R. rickettsii ω in chlamydiae. However, the analysis detected a possible sequence homolog of C. burnetii ω: namely, Wcw_0707, a hypothetical protein encoded by the genome of the Chlamydia-like organism Waddlia chondrophila (79) (Fig. 1A). Analysis of the sequence of Wcw_0707 revealed two features consistent with Wcw_0707 being an ω ortholog. First, Wcw_0707 is 107 amino acids long, similar in size to ω (~100 amino acids). Second, the Wcw_0707 N-terminal region (residues 7 to 62) exhibits strong sequence similarity to the C. burnetii ω (Fig. 1A) and Escherichia coli ω (Fig. 1B) N-terminal regions, which are known to be responsible for binding to the RNAP β′ subunit and facilitating the folding of β′ (14). We hypothesized that Wcw_0707 may be the ω subunit of W. chondrophila.

**FIG 2:**
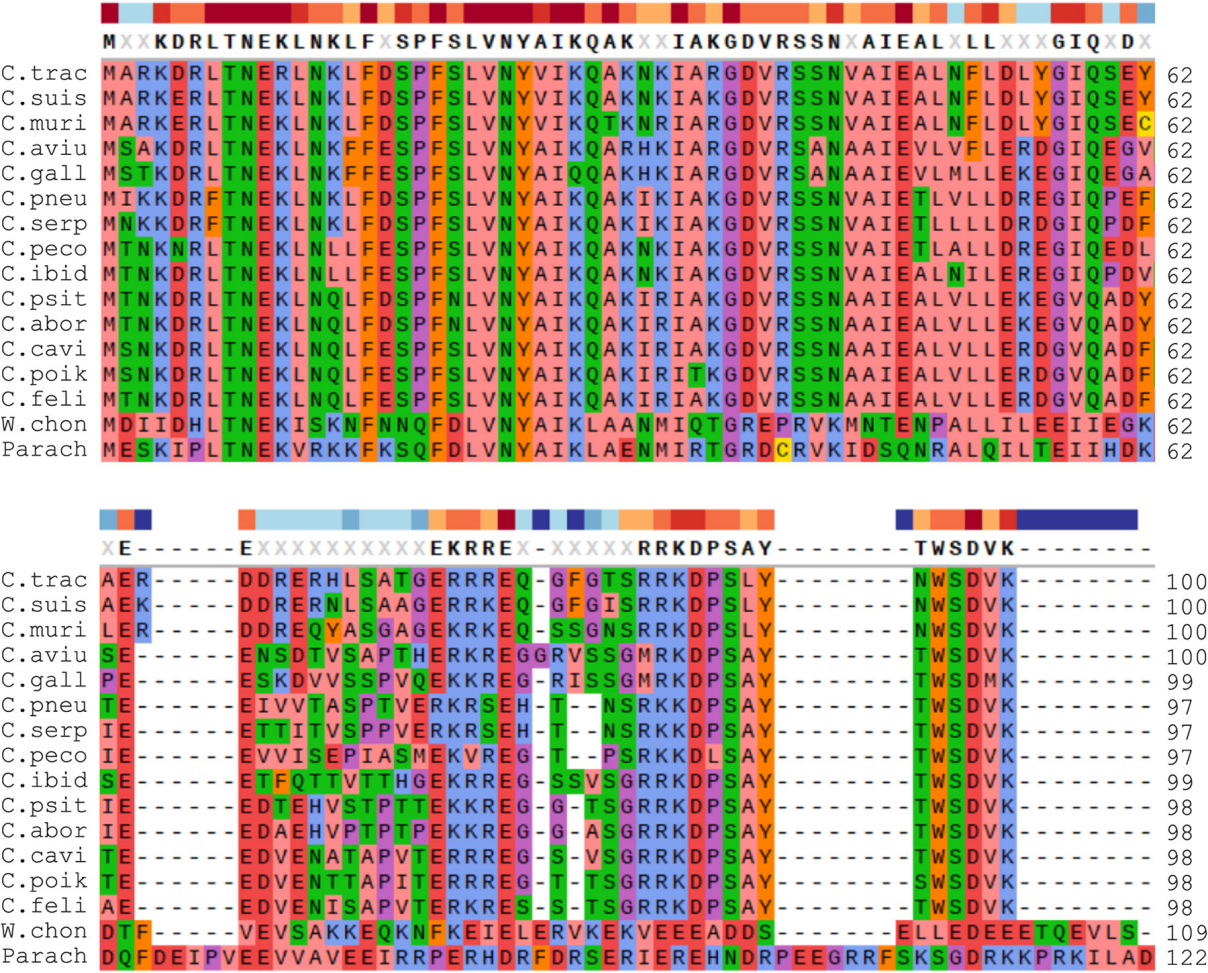
Sequence conservation among ω candidates in all vertebrate chlamydiae and Chlamydia-like organisms. The alignment was performed using ClustalX2. C. trac, Chlamydia trachomatis; C. suis, Chlamydia suis; C. muri, Chlamydia muridarum; C. aviu, Chlamydia avium; C. gall, Chlamydia gallinacea; C. pneu, Chlamydia pneumoniae; C. serp, Chlamydia serpentis; C. peco, Chlamydia pecorum; C. ibid, Chlamydia ibidis; C. psit, Chlamydia psittaci; C. abor, Chlamydia abortus; C. cavi, Chlamydia caviae; C. poik, Chlamydia poikilotherma; C. feli, Chlamydia felis; W. chon, Waddlia chondrophila; Parach, *Parachlamydiaceae*.

Given our primary interest in transcriptional regulation by the human sexually transmitted pathogen C. trachomatis, we next used Wcw_0707 as the query to search for a putative ω gene in the C. trachomatis genome. The search revealed strong sequence similarity between the N-terminal region of the hypothetical protein CTL0286 of C. trachomatis serovar L2 and the N-terminal region of Wcw_0707 of W. chondrophila (Fig. 1C). CTL0286 is a small protein of 100 amino acids, similar in length to previously reported RNAP ω subunits and Wcw_0707 (78). Notably, although CTL0286 exhibits only low overall sequence similarity to other reported bacterial ω subunits, it contains a key conserved set of amino acids found in ω subunits of a broad range of bacterial taxa (Fig. 1D). Additional BLASTP analyses using Wcw_0707 and CTL0286 as search queries identified a Wcw_0707 ortholog in a Chlamydia-like organism of the *Parachlamydiaceae* and identified CTL0286 orthologs in all vertebrate chlamydiae (Fig. 2; see also Table S1 in the supplemental material). Further analysis showed that the level of sequence variation of ω subunits among vertebrate chlamydiae is higher than that of 16S rRNA but lower than that of the major outer membrane protein (data not shown). These findings support the hypothesis that Wcw_0707 and its homolog are the ω subunits of the Chlamydia-like organisms W. chondrophila and *Parachlamydiaceae* and enable the hypothesis that CTL0286 and its homologs are the ω subunits of C. trachomatis and other vertebrate chlamydiae.

10.1128/mbio.03499-22.1 TABLE S1. AlphaFold and DisMeta analyses predict intrinsically disordered regions in the C-termini of the RNA polymerase ω subunits in vertebrate chlamydiae, Chlamydia-like organisms, and other obligate intracellular bacteria. Download Table S1, PDF file, 0.7 MB. Copyright © 2023 Cheng et al. 2023 Cheng et al. https://creativecommons.org/licenses/by/4.0/ This content is distributed under the terms of the Creative Commons Attribution 4.0 International license.

### Identification of chlamydial ω: synteny.

Upon manual examination of *rpoZ* in 10 bacterial genomes, we noted that the *rpoZ* gene in all cases is located immediately downstream of the *gmk* gene, which encodes guanylate kinase (Table 1). An *in silico* analysis identified *gmk-rpoZ* synteny in 18,302 of 23,517 fully sequenced bacterial genomes. The conservation of *gmk-rpoZ* synteny across the majority of bacterial taxa suggests that there is likely an adaptive advantage to *gmk-rpoZ* synteny, although the character of the adaptive advantage is not readily clear. In W. chondrophila and *Parachlamydiaceae*, the *wcw_0707* gene and its ortholog are located immediately downstream of the *gmk* gene, and in all vertebrate chlamydial species, the *ctl0286* gene and its orthologs also are located immediately downstream of *gmk* (Table 1). This conserved gene order provides further support for the hypothesis that WCW_0707, CTL0286, and their orthologs are chlamydial ω subunits.

**TABLE 1:** Conserved *gmk-rpoZ* linkages in bacterial genomes^*a*^

Bacterium	Gram stain result	Upstream gene	*rpoZ* or equivalent gene	Downstream gene
Bacillus anthracis	Positive	*gmk*	*rpoZ*	*coaBC*
Clostridium difficile	Positive	*gmk*	*rpoZ*	*coaBC*
Lactobacillus acidophilus	Positive	*gmk*	*rpoZ*	*priA*
Staphylococcus epidermidis	Positive	*gmk*	*rpoZ*	*SE0887*
Coxiella burnetii	Negative	*gmk*	*rpoZ*	*spoT*
Escherichia coli	Negative	*gmk*	*rpoZ*	*spoT*
Haemophilus influenzae	Negative	*gmk*	*rpoZ*	*spoT*
Vibrio cholerae	Negative	*gmk*	*rpoZ*	*spoT*
Gardnerella vaginalis	Variable	*gmk*	*rpoZ*	*dfp*
Mycobacterium tuberculosis	Variable	*gmk*	*rpoZ*	*metK*
**Waddlia chondrophila**	Negative	***gmk***	***wcw_0707***	*wcw_0708*
**Chlamydia trachomatis**	Negative	***gmk***	***ctl0286***	*metG*

a boldface for entries of highest relevance.

### Identification of chlamydial ω: predicted three-dimensional structural similarity.

**FIG 3:**
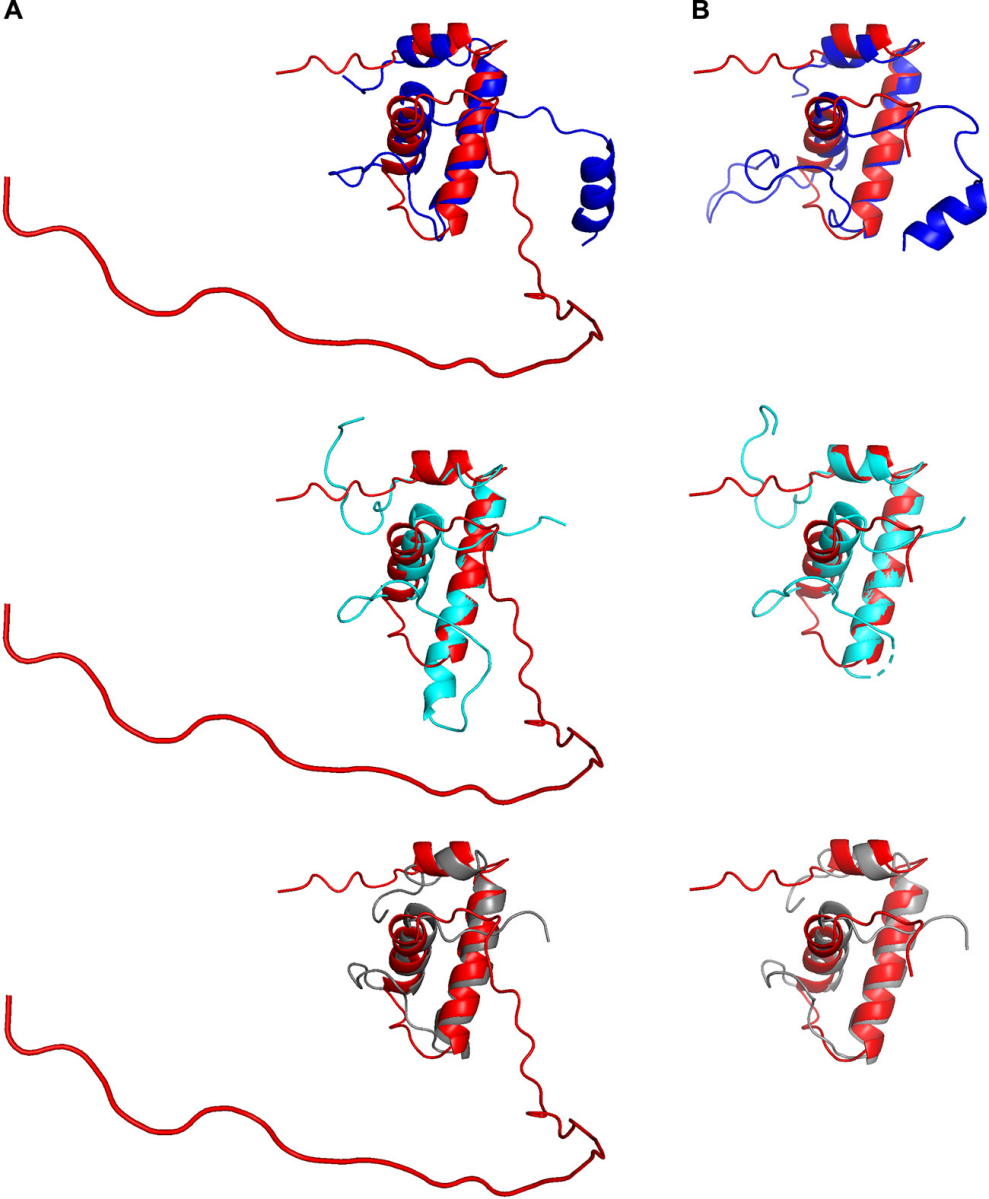
AlphaFold predictions for CTL0286. (A) Superimposition of the AlphaFold prediction for full-length CTL0286 (red) on experimental structures of Clostridium difficile, Mycobacterium tuberculosis, and Bacillus subtilis RNAP ω (blue, cyan, and gray, respectively). (B) Superimposition of AlphaFold prediction for the N-terminal region (residues 1 to 62) of CTL0286 (red) on experimental structures of Escherichia coli, Mycobacterium tuberculosis, and Bacillus subtilis RNAP ω (blue, cyan, and gray, respectively).

AlphaFold has recently become an indispensable resource for predicting the three-dimensional structures of proteins and protein complexes (84, 85). We first used AlphaFold to predict the three-dimensional structure of CTL0286. In the resulting predicted structure of CTL0286 (Fig. 3A), the N-terminal region (residues 1 to 58) contains three α helices (α1 [residues 9 to 15], α2 [residues 19 to 36], and α3 [residues 44 to 55]) that correspond to three α helices present in structurally characterized ω subunits (26, 86–96), and the C-terminal region (residues 58 to 100) is disordered. Three-dimensional-structure similarity searches of the AlphaFold prediction for full-length CTL0286, performed on the DALI server (97, 98), identified bacterial ω subunits as the three top hits (Fig. 3A), with Z-scores of 3.8, 3.4, and 3.1, for the RNAP ω subunits of Clostridium difficile (99), Mycobacterium tuberculosis (100), and Bacillus subtilis (101), respectively (Table 2). Three-dimensional-structure similarity searches of the AlphaFold prediction for the N-terminal region of CTL0286 (residues 1 to 62), performed on the DALI server (97, 98), identified bacterial ω subunits as the three top hits (Fig. 3B), with Z-scores of 5.2, 5.1, and 4.9 for the RNAP ω subunits of Escherichia coli (102), Mycobacterium tuberculosis (103), and Bacillus subtilis (104), respectively (Table 2).

**TABLE 2:** Proteins with structural homology to AlphaFold models of full-length CTL0286 or the N terminus of CTL0286^*a*^

Model	Rank	PDB accession no. (reference)	Protein	Bacterium	Z-score	RMSD
FL-CTL0286	1	7L7B (99)	RNAP ω	Clostridium difficile	3.8	3.9
	2	6BZO (100)	RNAP ω	Mycobacterium tuberculosis	3.4	3.1
	3	7CKQ (101)	RNAP ω	Bacillus subtilis	3.1	3.0

CTL0286(1–62)	1	5TJG (102)	RNAP ω	Escherichia coli	5.2	2.1
	2	6KOP (103)	RNAP ω	Mycobacterium tuberculosis	5.1	3.1
	3	7F75 (104)	RNAP ω	Bacillus subtilis	4.9	2.8

a Shown are proteins with structural homology to AlphaFold models of full-length CTL0286 (FL-CTL0286) or the N terminus of CTL0286 [CTL0286(1–62)]. The Z-score is an optimized similarity score defined as the sum of equivalent residue-wise C_α_-C_α_ distances between two proteins. RMSD, root mean square deviation of atomic positions.

**FIG 4:**
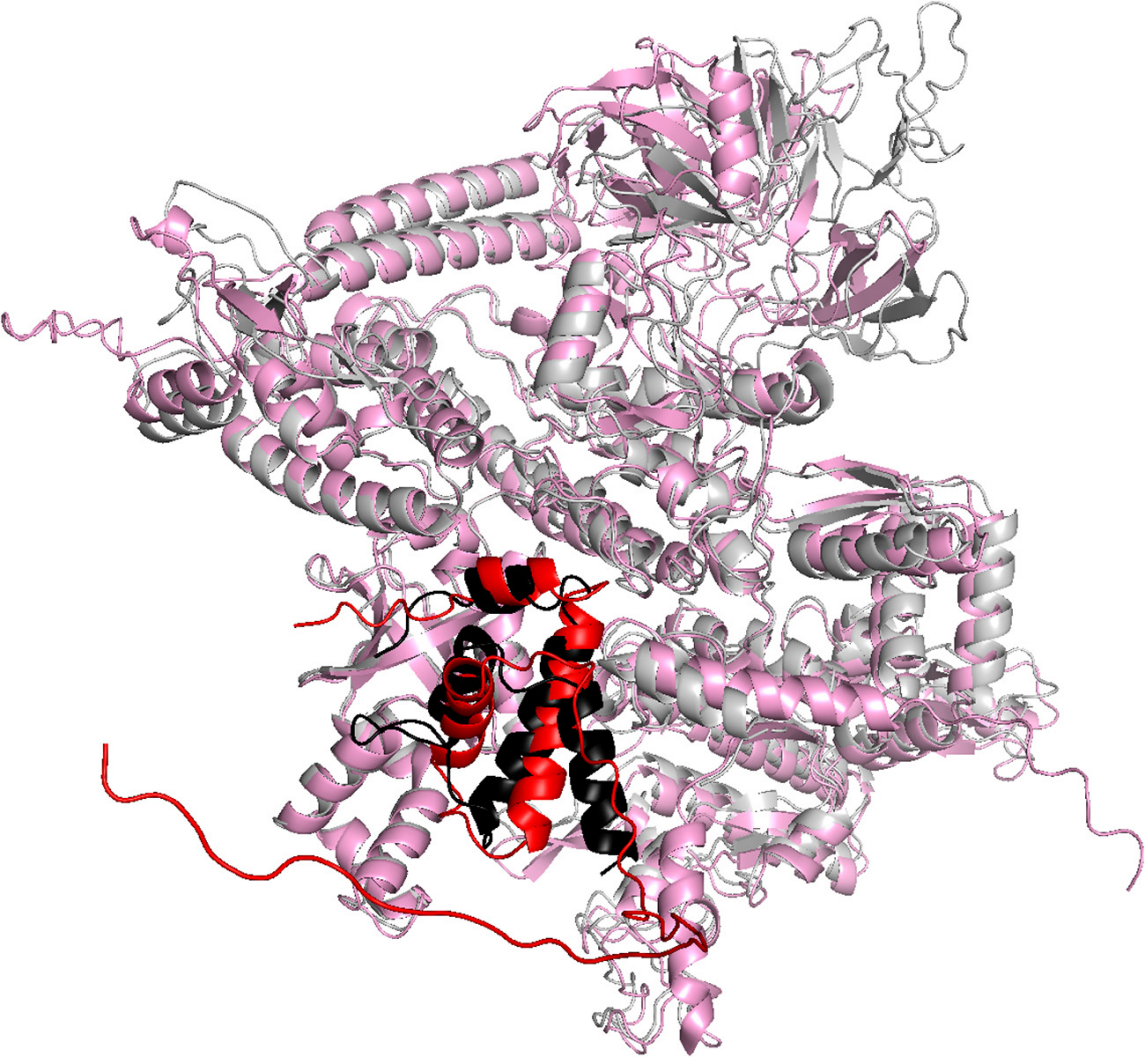
AlphaFold-Multimer predictions for the complex comprising CTL0286 and the C. trachomatis RNAP β′ subunit. Shown is a superimposition of the AlphaFold-Multimer prediction for CTL0286-β′ (red, CTL0286; pink, β′) on the experimental structure of E. coli RNAP (PDB accession number 6ALH) (black, ω; light gray, β′).

**FIG 5:**
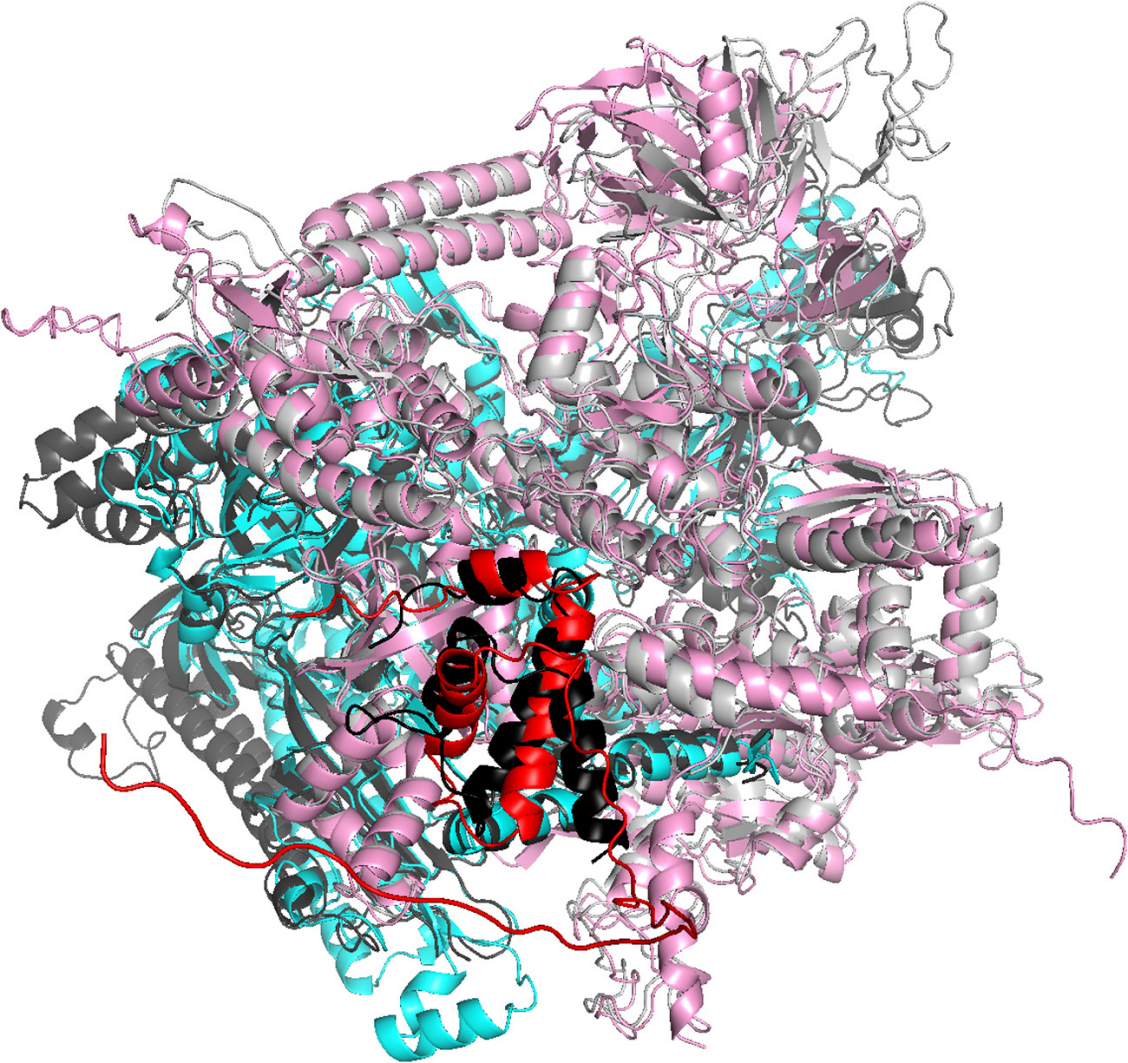
AlphaFold-Multimer predictions for the complex comprising CTL0286 and the C. trachomatis RNAP β′ and β subunits. Shown is a superimposition of the AlphaFold-Multimer prediction for CTL0286-β′-β (red, CTL0286; pink, β′; cyan, β) on the experimental structure of E. coli RNAP (PDB accession number 6ALH) (black, ω; light gray, β′; dark gray, β).

We next used AlphaFold-Multimer (85) to predict the three-dimensional structure of a complex of CTL0286 and the C. trachomatis RNAP β′ subunit. The resulting predicted three-dimensional structure of CTL0286-β′ was superimposable, with a root mean square deviation (RMSD) of 2.2 Å for CTL0286 and an RMSD of 4.0 Å for C. trachomatis RNAP β′ on a crystal structure of the ω-β′ subcomplex of the E. coli RNAP holoenzyme (PDB accession number 6ALH) (86) (Fig. 4). Significantly, the predicted three-dimensional structure of CTL0286-β′ includes interactions that bridge the RNAP β′ subunit N and C termini (Fig. 4), as observed in experimental structures of ω-containing RNAP and RNAP complexes (86, 105, 106), where they are believed to reduce the configurational entropy of the partly folded and folded states of the nearly 1,400-residue RNAP β′ subunits, thereby facilitating RNAP assembly and enhancing RNAP stability (10, 12, 14). We further used AlphaFold-Multimer to predict the three-dimensional structure of a heterotrimeric protein complex comprising CTL0286, C. trachomatis RNAP β′, and C. trachomatis RNAP β. The resulting predicted three-dimensional structure of CTL0286-β′ was superimposable, with RMSDs of 2.2 Å for CTL0286 and 2.7 Å for C. trachomatis RNAP β′ and β, on a crystal structure of the ω-β′-β subcomplex of the E. coli RNAP holoenzyme (PDB accession number 6ALH) (86) and includes interactions that bridge the N and C termini of β′ (Fig. 5).

Taken together, these findings provide further support for our hypothesis that CTL0286 and its orthologs are bona fide chlamydial ω subunits.

We next used AlphaFold to predict three-dimensional structures of the inferred ω orthologs of the 13 other vertebrate chlamydiae and two Chlamydia-like organisms (Table S1, column G). The AlphaFold-predicted three-dimensional structures of the inferred ω orthologs of vertebrate chlamydiae and Chlamydia-like organisms each contain a 55- to 62-residue N-terminal segment comprising a helical bundle superimposable on the helical bundle in experimental structures of bacterial RNAP ω subunits (88–96, 107) (Table S1, columns G and H). The ω orthologs of vertebrate chlamydiae have lengths of 97 to 100 residues and include C-terminal intrinsically disordered regions of 27 to 45 residues (Table S1, column G), and the ω orthologs of the Chlamydia-like organisms W. chondrophila and *Parachlamydiaceae* have lengths of 122 and 109 residues, respectively, and include C-terminal intrinsically disordered segments of 63 and 15 residues, respectively (Table S1, column G). In summary, the AlphaFold predictions suggest that the ω orthologs of the vertebrate chlamydiae and the two Chlamydia-like organisms contain not only the structurally conserved helical-bundle domain present in experimental structures of bacterial RNAP ω subunits but also intrinsically disordered C-terminal regions not present in experimental structures of other bacterial RNAP ω subunits (Table S1, columns G and H). DisMeta, a metaserver that predicts intrinsically disordered regions using seven different sequence-based predictors (108), also predicts highly disordered consensus sequences for the C-terminal segments of the ω orthologs of the vertebrate chlamydiae and the two Chlamydia-like organisms (Table S1, column I). We infer that the ω orthologs of vertebrate chlamydiae and Chlamydia-like organisms contain intrinsically disordered C-terminal regions not present in the experimental structures of other bacterial RNAP ω subunits (Table S1, columns 7 to 9).

### Identification of ω in other obligate intracellular bacteria.

**FIG 6:**
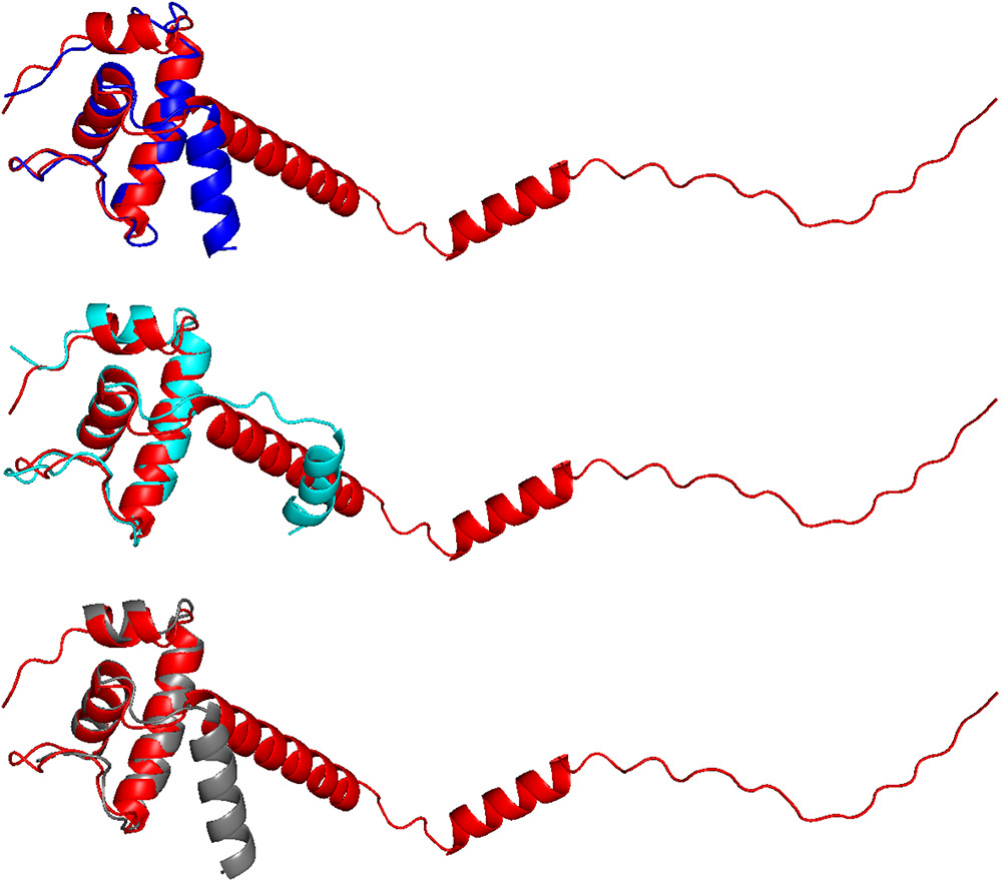
AlphaFold predictions for annotated ω of “*Candidatus* Midichloria” RNAP ω. Shown is a superimposition of the AlphaFold prediction for “*Candidatus* Midichloria” RNAP ω (red) on experimental structures of Pseudomonas aeruginosa, M. tuberculosis, and Xanthomonas oryzae RNAP ω (blue, cyan, and gray, respectively).

After the successful identification of the RNAP ω subunit in chlamydiae, we next determined if ω is present in other obligate intracellular bacterial taxa besides rickettsiae. NCBI searches identified annotated ω orthologs in the proteomes of *Anaplasma*, *Ehrlichia*, *Orientia*, *Wolbachia*, and *Candidatus* Midichloria. The pregenerated AlphaFold structural models of *Anaplasma*, *Ehrlichia*, *Orientia*, and *Wolbachia* ω orthologs in the UniProt database (www.uniprot.org) (109) each show a three-helix fold with three-dimensional structural similarity to experimentally determined structures of bacterial ω subunits, indicating that the annotations are likely correct. No pregenerated AlphaFold structural model of the annotated *Candidatus* Midichloria ω ortholog is available in the UniProt database (www.uniprot.org) (109). However, the generation of an AlphaFold structural model for the annotated *Candidatus* Midichloria ω ortholog (Fig. 6), followed by three-dimensional-structure similarity searches on the DALI server (97, 98), identified bacterial ω subunits as the three top hits, with Z-scores of 9.3, 8.6, and 8.6 for the RNAP ω subunits of Pseudomonas aeruginosa (110), Mycobacterium tuberculosis (100), and Xanthomonas oryzae (111), respectively, indicating that the annotation is likely correct (Table 3). We conclude that *Anaplasma*, *Ehrlichia*, *Orientia*, *Wolbachia*, and *Candidatus* Midichloria all possess RNAP ω subunits.

**TABLE 3:** Proteins with structural homology to the AlphaFold model of annotated *Candidatus* Midichloria RNAP ω

Rank	PDB accession no. (reference)	Protein	Bacterium	Z-score	RMSD
1	7XL3 (110)	RNAP ω	Pseudomonas aeruginosa	9.3	2.7
2	7L7B (100)	RNAP ω	Mycobacterium tuberculosis	8.6	3.0
3	6J9E (111)	RNAP ω	Xanthomonas oryzae	8.6	3.7

The ω subunits of *Rickettsia*, *Anaplasma*, *Ehrlichia*, *Orientia*, *Wolbachia*, and *Candidatus* Midichloria are longer than the ω subunits of most free-living bacteria (123 to 138 residues versus 78 to 107 residues) (Table S1). AlphaFold predicted long intrinsically disordered C-terminal segments (33 to 54 residues) in the ω subunits of *Rickettsia*, *Ehrlichia*, *Orientia*, and *Wolbachia* and intrinsically disordered subsegments in the C-terminal segments of the *Anaplasma* and *Candidatus* Midichloria ω subunits (38 to 41 residues) (Table S1). Consistent with the AlphaFold predictions, DisMeta also predicted highly disordered consensus sequences for the C-terminal segments of the ω subunits of *Rickettsia*, *Ehrlichia*, *Orientia*, *Wolbachia*, *Anaplasma*, and *Candidatus* Midichloria (Table S1). We infer that as for the ω subunits of vertebrate chlamydiae and *Chlamydia*-like organisms, the ω subunits of these other obligate intracellular bacteria tend to be longer than, and tend to contain intrinsically disordered C-terminal regions not present in, the experimental structures of other bacterial RNAP ω subunits (Table S1).

### Absence of ω in *Mycoplasma* and *Ureaplasma*.

We next extended our RNAP ω subunit search to the facultative intracellular bacterium Mycoplasma genitalium, whose 580-kb genome is the smallest known bacterial genome (112). An NCBI search failed to identify an annotated *rpoZ* gene in M. genitalium. Interestingly, our search also failed to identify an annotated *rpoZ* gene in other *Mycoplasma* species, even though most have genome sizes comparable to that of Chlamydia. Our search also failed to identify an annotated *rpoZ* gene in *Ureaplasma* (113), which is phylogenetically closely related to *Mycoplasma*. To verify the absence of ω subunits in these organisms, we first checked the gene immediately downstream of the *gmk* gene in *Mycoplasma* for possible sequence similarity to *rpoZ*, and we found none (112, 113). We next performed AlphaFold modeling for all 68 hypothetical proteins of Mycoplasma pneumoniae having sizes comparable to those of bacterial ω subunits (i.e., sizes of 40 to 150 amino acids) (114). AlphaFold predicted multi-α-helix folds for 26 of the 68 proteins. Three-dimensional-structure similarity searches of these 26 AlphaFold predictions, performed on the DALI server (97, 98), failed to identify structures of experimentally determined bacterial ω subunits as possible matches. We infer that *Mycoplasma* and *Ureaplasma* are unlikely to have RNAP ω subunits.

## DISCUSSION

In this report, we present multiple lines of evidence for the existence of an RNAP ω subunit in chlamydiae. Although a lack of strong, continuous sequence homology had previously precluded the identification of a chlamydial ω subunit, multistep BLASTP analysis led to the identification of CTL0286 as a candidate (Fig. 1). Like *rpoZ* in the supermajority of bacteria, *ctl0286* is located immediately downstream of *gmk* (Table 2 and data not shown). The AlphaFold-predicted three-dimensional structures of CTL0286 exhibit strong similarities to the experimental three-dimensional structures of the ω subunits of a broad range of bacterial taxa (29, 101, 111, 115, 116). The AlphaFold-Multimer-predicted three-dimensional structures of complexes of CTL0286 with the C. trachomatis RNAP β′ subunit and of CTL0286 with the C. trachomatis RNAP β′ and β subunits exhibit strong similarity to the experimental three-dimensional structures of ω-β′ and β′-β complexes (Fig. 4 and 5) (86, 87). The identification of CTL0286 as the C. trachomatis ω subunit demonstrates the power of the use of combinations of sequence similarity analysis, synteny analysis, and AlphaFold and AlphaFold-Multimer analyses for identifying proteins “missing” from proteomes and annotating functions of hypothetical proteins in proteomes.

Our extended analysis further showed that like Chlamydia, other obligate intracellular bacteria (i.e., *Rickettsia*, *Anaplasma*, *Ehrlichia*, *Orientia*, *Wolbachia*, and *Candidatus* Midichloria) also encode ω orthologs (Fig. 6 and data not shown), but the facultative intracellular bacteria *Mycoplasma* and *Ureaplasma* do not. Together with previous findings demonstrating the existence of ω orthologs in archaea and eukaryotes (27–29), these findings suggest that all living organisms from bacteria to humans have ω orthologs, with *Mycoplasma* and *Ureaplasma* likely being the only exceptions.

ω plays roles in σ-RNAP core enzyme associations (21–23) and thereby influences promoter recognition selectivity (21–23). Chlamydiae possess a principal σ factor and two alternative σ factors (77, 78, 117). The principal σ factor, σ^66^, is involved in the transcription of most chlamydial genes throughout the developmental cycle; the alternative σ factors, σ^28^ and σ^54^, are required for the expression of certain late genes (118–120). The different chlamydial σ factors also differentially affect responses to stress conditions (71, 76). It would be equally interesting to investigate if and how the chlamydial ω subunit regulates the σ-RNAP core enzyme association in chlamydial developmental stages and in response to various stress conditions.

In summary, we have identified the long-missing ω subunit of the cRNAP. As with most scientific studies, this discovery raises more questions than it answers. There is a need to determine whether the chlamydial ω subunit plays solely a structural role in cRNAP assembly and stability or whether it also functions in the regulation of chlamydial growth, development, and stress responses.

## MATERIALS AND METHODS

### BLASTP analysis.

Web-based BLASTP analysis (https://blast.ncbi.nlm.nih.gov/Blast.cgi?PAGE=Proteins) was performed using default settings (83). Multiple-protein-sequence alignment was performed with ClustalX2 on a Windows computer (121) using default settings (122, 123).

### Three-dimensional-structure prediction.

AlphaFold version 2.2.0 (84) was installed locally and run using the reduced-database option with a maximum template date of 1 November 2021 and the multimer preset enabled for the cRNAP β′-CTL0286 and cRNAP β-β′-CTL0286 complex predictions. The multimer predictions were run with the default pretrained AlphaFold-Multimer models (85), and the zero-ranked predictions (i.e., those with the lowest predicted local distance difference test [pLDDT] scores) were used for the figures for each complex. CTL0286 monomer structure prediction was performed with the default monomer preset and the full database option using the original CASP14 monomer models without ensembling. Model prediction and amber relaxation were performed for all predictions using a single Nvidia Tesla V100 Volta GPU with 16 GB of memory. Since the total sequence lengths significantly increase the space complexity, forced unified memory was enabled, and the accelerated linear algebra (XLA) memory fraction environmental variable was set to 4.0 to avoid out-of-memory errors during the run time.

### Three-dimensional-structure similarity analysis.

The structural homology search for the AlphaFold model of CTL0286 was performed using the DALI server heuristic PDB search option (97, 98) (available at http://ekhidna2.biocenter.helsinki.fi/dali/). PDB90, a nonredundant subset of the PDB at 90% sequence identity, was used.

### High-throughput synteny analysis.

CSBFinder-S (v0.6.3) (124) was used with the default settings to find the *gmk-rpoZ* synteny across 23,517 fully sequenced bacterial genomes downloaded from the NCBI genome database.

DeepNOG (v1.2.3) (125) was run using the default settings to obtain the COG (Clusters of Orthologous Genes) identification for each gene. Strand information was obtained from the corresponding genomic .gff file for every genome downloaded. The *gmk-rpoZ* synteny was identified by finding COG0194 (*gmk*) and COG1758 (*rpoZ*) together within the CSBFinder-S output.

### Prediction of intrinsically disordered regions.

Prediction of intrinsically disordered regions. The DisMeta server (108) was used to predict intrinsically disordered regions. Regions with a 3.5 disordered consensus of 7 disorder predictors were deemed intrinsically disordered regions.
