# Supplementary material for: Identification and Structural Modeling of the RNA Polymerase Omega Subunits in Chlamydiae and Other Obligate Intracellular Bacteria
Source: mBio. 2023 Jan 31;14(1):e03499-22. doi: 10.1128/mbio.03499-22 (PMC9973325; doi:10.1128/mbio.03499-22)
Supplement: TABLE S1. [file mbio.03499-22-s0001.pdf]

Table S1. AlphaFold and Dismeta analyses predict intrinsically disordered regions in the C-termini of the RNA polymerase omega subunits in vertebrate chlamydiae, *Chlamydia* -like organisms, and other obligate intracellular bacteria.

Species	Accession	Weblink	Sequence	Length	AlphaFold-modeled structure and description	X-ray crystal or cryoEM structure and description	Dismeta consensus of 7 disorder predictors
**Vertebrate chlamydiae**							
C. trachomatis	CAP03725.1	https://www.	MARKDRLTNERLNKL FDSPFSLVNYVIKQA KNKIARGDVRSSNVA IEALNFLDLYGIQSE YAERDDRERHLSATG ERRREQGFGTSRRKD PSLYNWSDVK	100	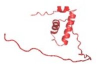 C-terminal IDR: 56-100	NA (not applicable)	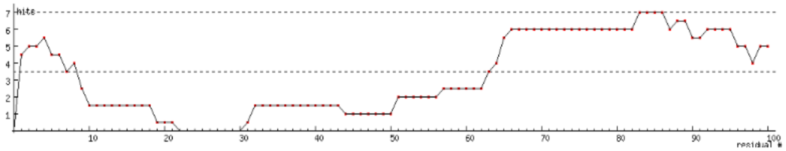
C. suis	WP_080121954.1	https://www.	MARKERLTNEKLNKL FDSPFSLVNYVIKQA KNKIAKGDVRSSNVA IEALNFLDLYGIQSE YAEKDDRERNLSAAG ERRKEQGFGISRRKD PSLYNWSDVK	100	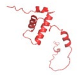 C-terminal IDRs: 56-65, 74-100	NA	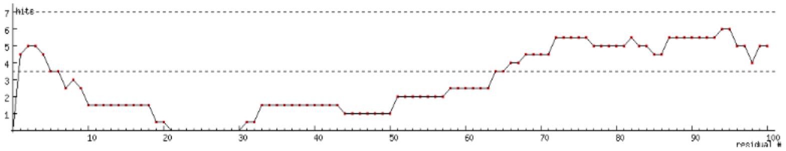
C. muridarum	WP_010230088.1	https://www.	MARKERLTNEKLNKL FDSPFSLVNYVIKQT KNRIARGDVRSSNVA IEALNFLDLYGIQSE CLERDDREQYASGAG EKRKEQSSGNSRRKD PSLYNWSDVK	100	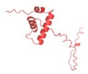 C-terminal IDR: 56-100	NA	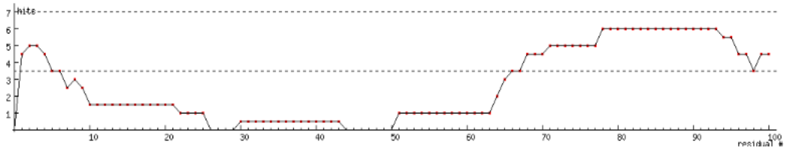
C. ibidis	WP_020370251.1	https://www.	MTNKDRLTNEKLNLL FESPFSLVNYAIKQA KNKIAKGDVRSSNVA IEALNILEREGIQPD VSEETFQTTVTTHGE KRREGSSVSGRRKDP SAYTWSDVK	99	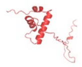 C-terminal IDR: 56-99	NA	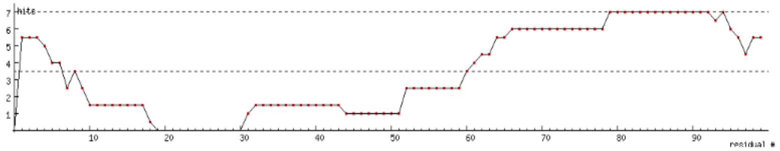
C. psittaci	WP_014946110.1	https://www.	MTNKDRLTNEKLNQL FDSPFNLVNYVIKQA KIRIAKGDVRSSNAA IEALVLLEKEGVQAD YIEEDTEHVSTPTTE KKREGGTSGRRKDPS AYTWSDVK	98	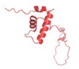 C-terminal IDR: 56-98	NA	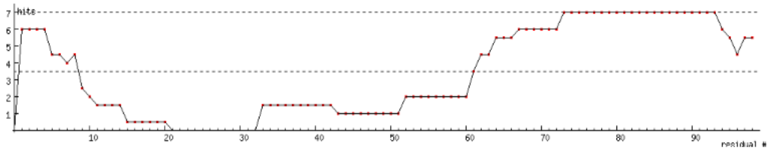
C. abortus	WP_139415256.1	https://www.	MTNKDRLTNEKLNQL FDSPFNLVNYAIKQA KIRIAKGDVRSSNAA IEALVLLEKEGVQAD YIEEDAEHVPTPTPE KKREGGASGRRKDPS AYTWSDVK	98	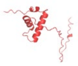 C-terminal IDR: 56-98	NA	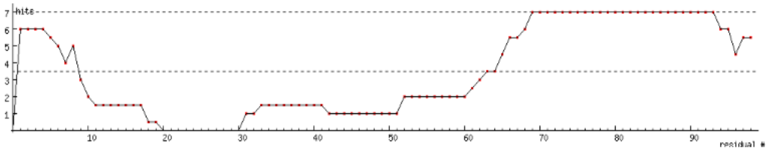

C. caviae	WP_011006609.1	https://www.ı	MSNKDRLTNEKLNQL FESPFSLVNYAIKQA KIRIAKGDVRSSNAA IEALVLLERDGVQAD FTEEDVENATAPVTE RRREGSVSGRRKDPS AYTWSDVK	98	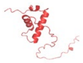 C-terminal IDR: 56-98	NA	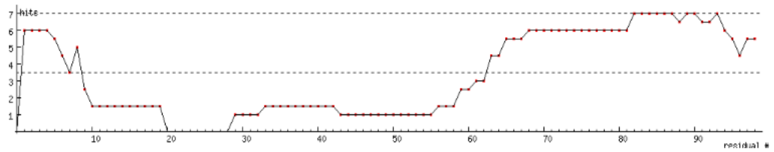
C. pneumoniae	WP_010882771.1	https://www.ı	MIKKDRFTNEKLNKL FDSPFSLVNYAIKQA KIKIAKGDVRSSNVA IETLVLLDREGIQPE FTEEIVVTASPTVER KRSEHTNSRKKDPSA YTWSDVK	97	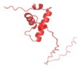 C-terminal IDR: 56-97	NA	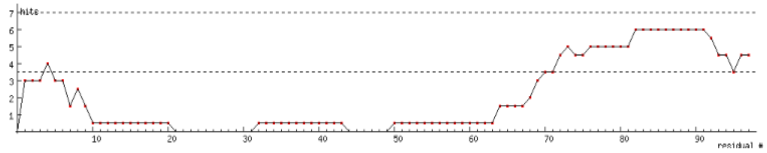
C. poikilotherma	WP_117274417.1	https://www.ı	MSNKDRLTNEKLNQL FESPFSLVNYAIKQA KIRITKGDVRSSNAA IEALVLLERDGVQAD FTEEDVENTTAPITE RRREGTTSGRRKDPS AYSWSDVK	98	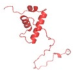 C-terminal IDR: 56-98	NA	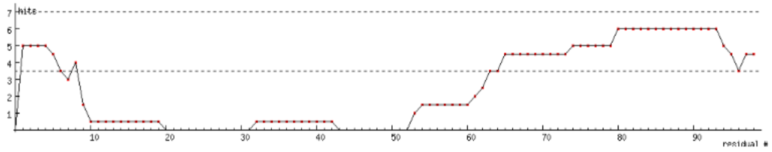
C. felis	WP_011457911.1	https://www.ı	MTNKDRLTNEKLNQL FESPFSLVNYAIKQA KIRIAKGDVRSSNAA IEALVLLERDGVQAD FAEEDVENISAPVTE RKRESSTSGRRKDPS AYTWSDVK	98	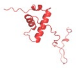 C-terminal IDR: 56-98	NA	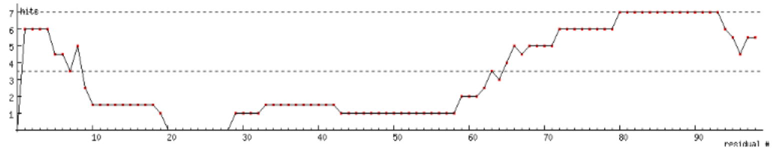
C. serpentis	WP_108896272.1	https://www.ı	MNKKDRFTNEKLNKL FDSPFSLVNYAIKQA KIKIAKGDVRSSNVA IETLLLLDRDGIQPD FIEETTITVSPPVER KRSEHTNSRKKDPSA YTWSDVK	97	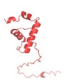 C-terminal IDR: 56-97	NA	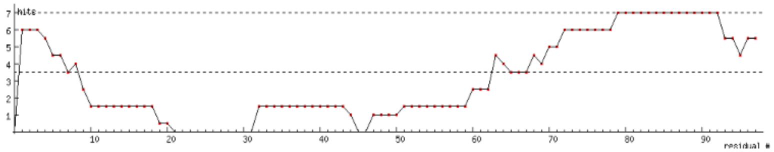
C. avium	WP_038500557.1	https://www.ı	MSAKDRLTNEKLNKF FESPFSLVNYAIKQA RHKIARGDVRSANAA IEVLVFLERDGIQEG VSEENSDTVSAPTHE RKREGGRVSSGMRKD PSAYTWSDVK	100	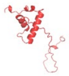 C-terminal IDR: 56-100	NA	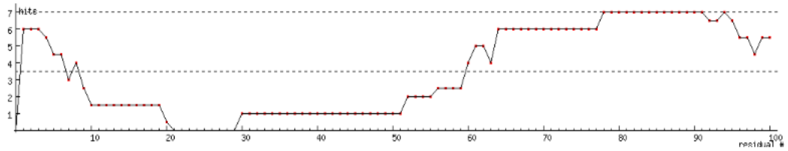
C. gallinacea	WP_021828442.1	https://www.ı	MSTKDRLTNEKLNKF FESPFSLVNYAIQQA KHKIARGDVRSANAA IEVLMLLEKEGIQEG APEESKDVVSSPVQE KKREGRISSGMRKDP SAYTWSDMK	99	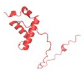 C-terminal IDR: 56-99	NA	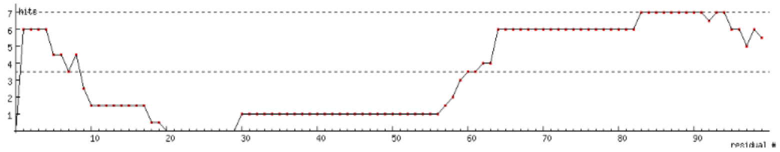

C. pecorum	WP_013713021.1	https://www.ı	MTNKNRLTNEKLNLL FESPFSLVNYAIKQA KNKIAKGDVRSSNVA IETLALLDREGIQED LIEEVVISEPIASME KVREGTPSRKKDLSA YTWSDVK	97	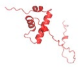 C-terminal IDR: 56-97	NA	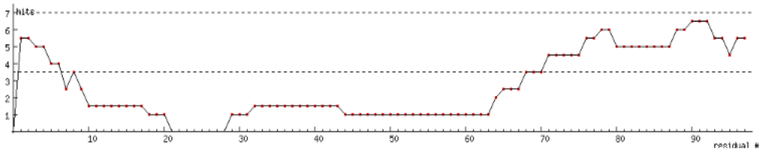

***Chlamydia*-** **like organisms**

Waddlia chondrophila	WP_013181793.1	https://www.ı	MDIIDHLTNEKISKN FNNQFDLVNYAIKLA ANMIQTGREPRVKMN TENPALLILEEIIEG KDTFVEVSAKKEQKN FKEIELERVKEKVEE EADDSELLEDEEETQ EVLS	109	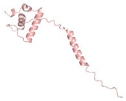 C-terminal IDR: 60-76, 96-109	NA	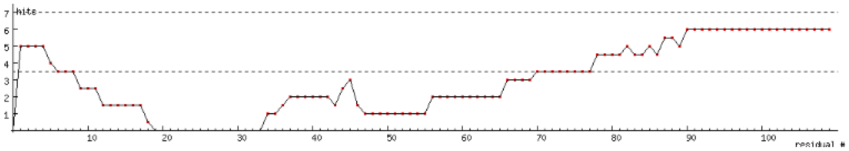
Parachlamydiaceae	MBA3816900.1	https://www.ı	MESKIPLTNEKVRKK FKSQFDLVNYAIKLA ENMIRTGRDCRVKID SQNRALQILTEIIHD KDQFDEIPVEEVVAV EEIRRPERHDRFDRS ERIEREHNDRPEEGR RFSKSGDRKKPRKIL AD	122	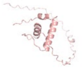 C-terminal IDR: 60-122	NA	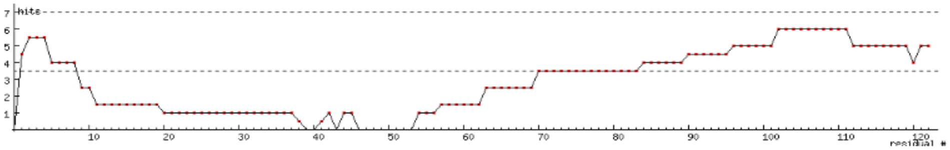

Other obligate intracellular bacteria

Rickettsia rickettsii	WP_012151045	https://www.ı	MARITAEDCNKIIPD RFRLVVLATRYAKLL NYKVETNHIKKEKLD KPPVIALRRIAAGKV SVAQLEQDLINSLRT RTMIEPLVNQDESEA VEEKFEYLPEVYIGE DYSDLDDQIFIDEHG EDYETDK	127	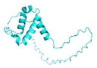 C-terminal IDR: 74-127	NA	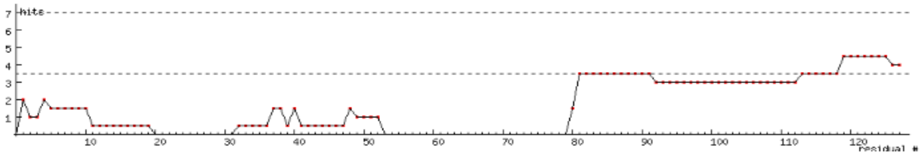
Anaplasma marginale	ACM49195.1	https://www.ı	MREGDSGYFSCYDGN RFRLVILASQRAHEL SSGACTAVARKGDKN TVVALREIVGEQLDL AAVFKLAVNRCRKYL EEFTNAREVAAARSS QAAPKSAPGQEIGKS FREKDPSAAAFLDQE QFFSGGGE	128	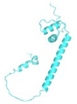 C-terminal IDRs: 91-111, 124-128	NA	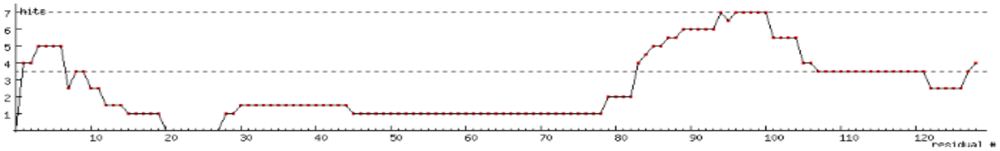
Ehrlichia minasensis	CEI84681.1	https://www.ı	MTRLTVEECMGRTNN KFKLVILASQRSHDL NSGACPVIKHKNSKN TVIALKEIAAKQLDV ASLFNLSVQRCRKYM EKFINSDEQYVANKA KIDIFQQNAIASNFN ELGNNSNNQNTNLLG RDNFFSTPENRNTSN TDS	138	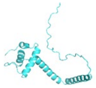 C-terminal IDR: 105-138	NA	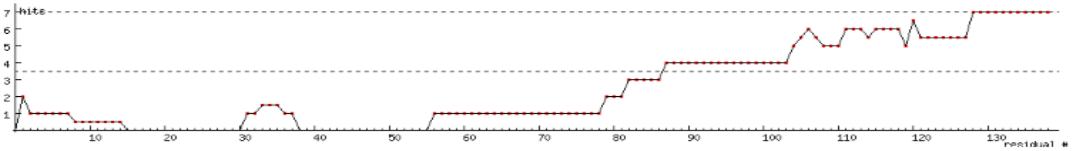
Orientia tsutsugamushi	SPR15298.1	https://www.ı	MANTITHYPQHVANR FELVSLSAHLVIELN SGSNIMASHAKKDSN LIMTALNGILTEEVN IPELREKLILKHQTQ SHNYLSKLQKNKFNN DQEGYFNNFESDSEF DNQQIYLSSDNLSII NDDDDTRKI	129	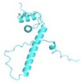 C-terminal IDR: 90-129	NA	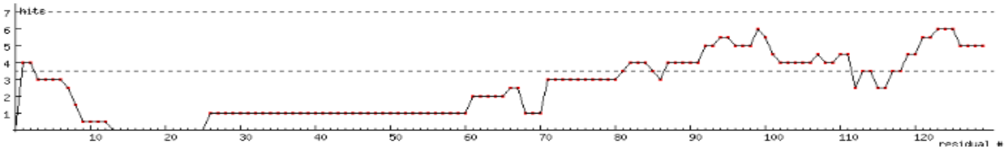

Wolbachia endosymbiont of Armadillidium vulgare str. wVulC	KLT22225.1	https://www.ı	MVESIVEKCVEQVHN<br>RFKLVLLASQRTHDL<br>STGTSDPVQMVKFKG<br>HKDTIVALYEIAEKK<br>VDTHELFNLLVKRCK<br>EYMKGNMNNAYSRSP<br>SKLASLLNFSDHQFN<br>TDLDISQGSQDDEID<br>DQDSGEEVPI	130	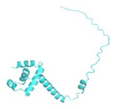<br>C-terminal IDR: 81-90, 98-130	NA	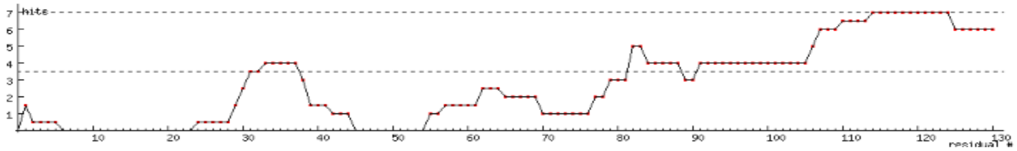
Candidatus Midichloria mitochondrii	WP_013950554.1	https://www.ı	MARITIEGCLTKVED<br>RFALVVLAAQRARDL<br>AFGATPLVPKNNDKY<br>AVIALREIADSALDT<br>KSLQEAIIKRNQRKQ<br>LLQVEDQYETVDEET<br>ESAYQQAMANFAVPS<br>RRKHSSNDDEDVDDE<br>TGD	123	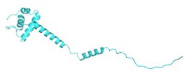<br>C-terminal IDRs: 83-88, 102-123	NA	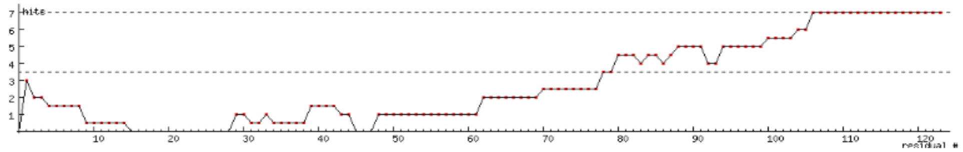
**<u>Bacteria with structurally defined RNAP ω subunits</u>**							
E. coli	6PSW_K	https://www.ı	MARVTVQDAVEKIGN<br>RFDLVLVAARRARQM<br>QVGGKDPLVPEENDK<br>TTVIALREIEEGLIN<br>NQILDVRERQEQQEQ<br>EAAELQAVTAIAEGR<br>R	91	ND (not determined)	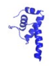<br>Helices: 7-14, 17-32, 45-56, 62-80	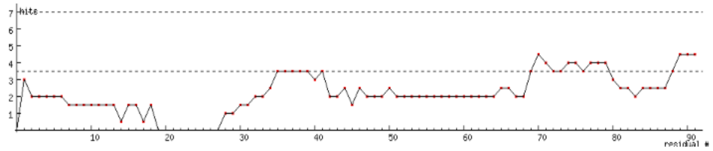
	3LU0_E	**https://www.**	MARVTVQDAVEKIGN<br>RFDLVLVAARRARQM<br>QVGGKDPLVPEENDK<br>TTVIALREIEEGLIN<br>NQILDVRERQEQQEQ<br>EAAELQAVTAIAEGR<br>R	91	ND	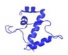<br>Helices: 5-13, 15-33, 50-56, 74-85	
	4MEY_E	<u>https://www.ı</u>	MARVTVQDAVEKIGN<br>RFDLVLVAARRARQM<br>QVGGKDPLVPEENDK<br>TTVIALREIEEGLIN<br>NQILDVRERQEQQEQ<br>EAAELQAVTAIAEGR<br>R	91	ND	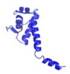<br>Helices: 5-13, 16-33, 44-56, 60-70, 82-90	
Clostridium difficile	7L7B_E	https://www.ı	MLKPSINEVLEKIDN<br>RYYLVGTVSKRARKL<br>IDGEEPYVSNKTKEK<br>PVCVATKEVASGKIT<br>YRLLTEEEIEIEEAR<br>HHAEQHQQISEEE	91	ND	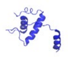<br>Helices: 15-32, 45-57, 65-77	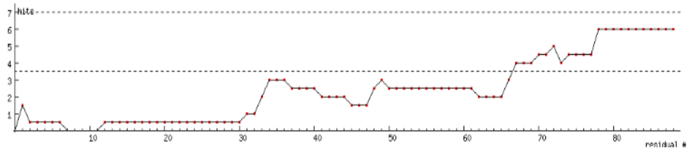
Francisella tularensis	6WMP_E	https://www.ı	MARVTVEDCLDKVET<br>RFDLVVLASMRANKI<br>LKNGYSESMENEKKE<br>KATVVALREIAESEI<br>TSEQILRNEIEG	72	ND	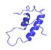<br>Helices: 17-32, 47-56	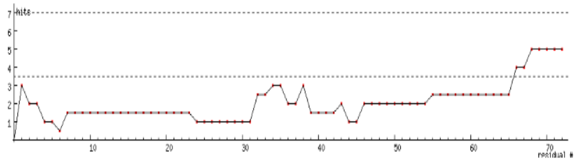

Mycobacterium tuberculosis	6DCF_E	https://www.ı	MSTPHADAQLNAADD<br>LGIDSSAASAYDTPL<br>GITNPPIDELLSRAS<br>SKYALVIYAAKRARQ<br>INDYYNQLGDGILEY<br>VGPLVEPGLQEKPLS<br>IALREIHGDLLEHTE<br>GE	107	ND	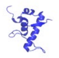	Helices: 30-34, 36-42, 46-67, 87-98	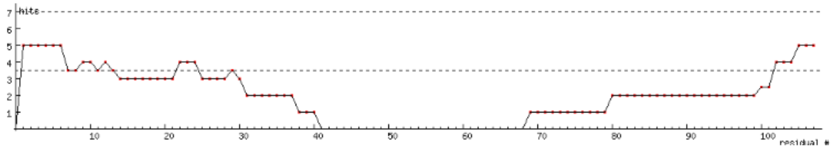
	6VVT_E	https://www.ı	MSTPHADAQLNAADD<br>LGIDSSAASAYDTPL<br>GITNPPIDELLSRAS<br>SKYALVIYAAKRARQ<br>INDYYNQLGDGILEY<br>VGPLVEPGLQEKPLS<br>IALREIHGDLLEHTE<br>GE	107	ND	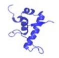	Helices: 30-34, 36-42, 46-66, 87-98	
Pseudomonas aeruginosa	7XYA_E	https://www.ı	MARVTVEDCLDNVDN<br>RFELVMLATKRARQL<br>ATGGKEPKVAWENDK<br>PTVVALREIASGLVD<br>ENVVQQEDIVEDEPL<br>FAAFDDEANTEAL	88	ND	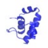	Helices: 8-14, 16-32, 45-56, 60-68	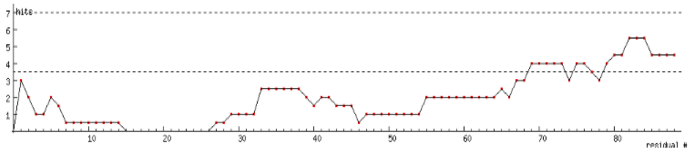
Staphylococcus aureus	6VJS_E	https://www.ı	MARVTVQDAVEKIGN<br>RFDLVLVAARRARQM<br>QVGGKDPLVPEENDK<br>TTVIALREIEEGLIN<br>NQILDVRERQEQQEQ<br>EAAELQAVTAIAEGR<br>R	91	ND	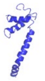	Helices: 16-31, 46-56, 61-79, 80-90	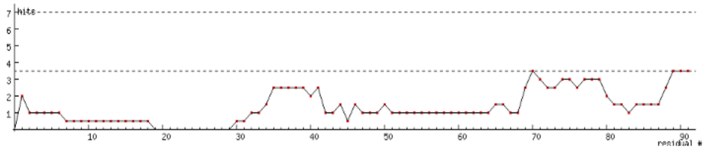
Streptomyces coelicolor	7VPZ_E	https://www.ı	MSSSISAPEGIINPP<br>IDELLEATDSKYSLV<br>IYAAKRARQINAYYS<br>QLGEGLLEYVGPLVD<br>THVHEKPLSIALREI<br>NAGLLTSEAIEGPAQ	90	ND	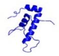	Helices: 16-24, 27-45, 68-77	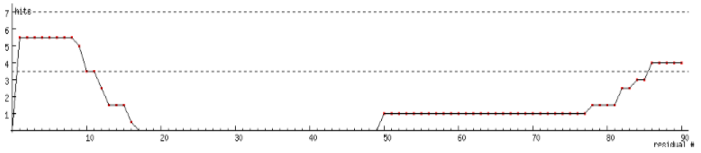
Thermus aquaticus	1I6V_E	https://www.ı	MAEPGIDKLFGMVDS<br>KYRLTVVVAKRAQQL<br>LRHRFKNTVLEPEER<br>PKMRTLEGLYDDPNA<br>VTWAMKELLTGRLFF<br>GENLVPEDRLQKEME<br>RLYPTEEEA	99	ND	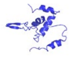	Helices: 6-13, 18-29, 61-69, 81-89	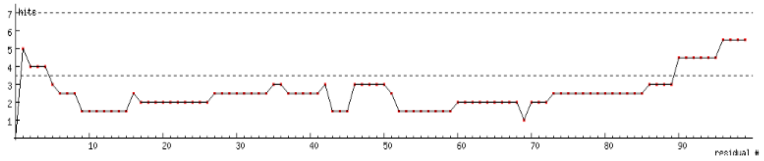
Thermus thermophilus	4GZY_E	https://www.ı	MAEPGIDKLFGMVDS<br>KYRLTVVVAKRAQQL<br>LRHGFKNTVLEPEER<br>PKMQTLEGLFDDPNA<br>VTWAMKELLTGRLVF<br>GENLVPEDRLQKEME<br>RLYPVEREE	99	ND	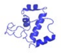	Helices: 5-11, 17-33, 61-68, 82-91	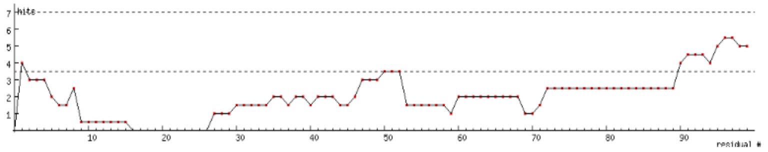

	4GZZ_E	https://www.r	MAEPGIDKLFGMVDS KYRLTVVVAKRAQQL LRHGFKNTVLEPEER PKMQTLEGLFDDPNA VTWAMKELLTGRLVF GENLVPEDRLQKEME RLYPVEREE	99	ND	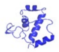 Helices: 5-11, 17-33, 61-68, 82-91	
Xanthomonas oryzae	6J9F_E	https://www.r	MARITVEDCLEVVNN RFELVMMASKRARQL ANGVQPLIENAAASD KPTVMALREIAARRI DNALIDEVEKAERER AEREALEWAAAEVVA DEDMSKNDD	99	ND	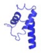 Helices: 6-27, 48-57, 62-74	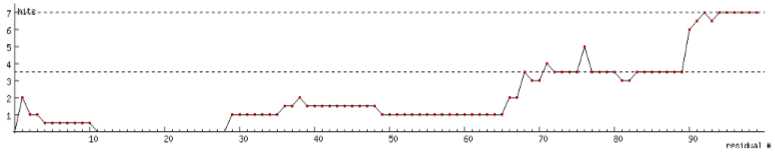
